# Influencing the selectivity of grafted anion exchangers utilizing the solubility of the radical initiator during the graft process

**DOI:** 10.1016/j.acax.2019.100019

**Published:** 2019-05-23

**Authors:** Achim Kaltz, Lea Bohra, Jonathan S. Tripp, Andreas Seubert

**Affiliations:** University of Marburg, Faculty of Chemistry, Analytical Chemistry, Hans-Meerwein-Str. 4, 35032, Marburg, Germany

**Keywords:** Ion chromatography, Grafting, Graft-polymerization, Synthesis, Anion exchangers, Selectivity

## Abstract

A previously published radical graft-functionalization method for the synthesis of high performance anion exchangers was further investigated to control the capacity and selectivity of the exchangers. Using a hydrophobic radical initiator instead of a hydrophilic one diminished the influence of rivaling homopolymerization of monomer during the functionalization step. Instead of only generating monomer radicals in free solution the radicals are ideally generated on top of the PS/DVB surface. However, in both cases the selectivity factors of polarizable anions bromide and nitrate in relation to chloride increased strongly with increasing capacity of the exchanger. Higher exchanger capacities could lead to coelution of bromide and/or nitrate with other analytes such as sulfate or phosphate when using the eluent as proposed in this work. By variation of the organic solvent used for functionalization it was possible to remove both the rivaling homopolymerization and the strong influence of the capacity on the selectivity. With increasing solubility of the hydrophobic radical initiator in the organic solvent the influence of the homopolymerization and the influence on the selectivity factor of bromide and nitrate decreased. Additionally, a change of bromate selectivity factor could be observed. The bromate signal is shifted closer towards the chloride signal. However, with increasing solubility of the radical initiator in the organic solvent the observed capacity of the exchangers decreases linearly, resulting in higher amounts of monomer needed for functionalization.

## Highlights

• Anion exchangers have been prepared using free radical graft polymerization and a molecularly predefined ion exchange site.
• Solubility of the radical initiator in the graft solvent mixture influences the capacity and ion exchange selectivity.
• The conversion rate of remaining double bonds of highly crosslinked DVB polymers explain selectivity and performance changes.

## Graphical abstract

**Figure:**
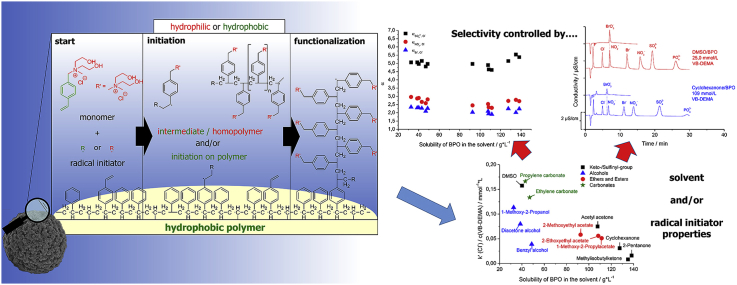

## 1 Introduction

The basis of modern ion chromatography (IC) was developed by Small et al. in 1975 [1] and by Gjerde, Fritz and Schmuckler in the following years [[2], [3], [4]]. Their work allowed IC to become the most beneficial tool for analysis of a wide range of inorganic and small organic ions as it is today. Current research in IC focuses on the development of new applications for existing stationary phases and on the development of new stationary phases for challenging applications. New IC stationary phases should offer good chromatographic performance, fast separations and a specific selectivity to solve analytical problems [[5], [6], [7], [8]]. The control of selectivity is a difficult task, highly depending on the construction principle of the stationary phase [[9], [10], [11]]. Therefore several commercial columns feature a very specific area of application and utilize a variety of supporting materials and functionalization methods [[12], [13], [14]].

Typically, stationary phases are made by functionalization of a supporting material with a functional group or a functional layer containing the ion exchange groups. The supporting materials usually consist of organic polymers like polyvinylalcohols [15,16], polymethacrylates [17,18], ethylvinylbenzene-divinylbenzene (EVB-DVB) or polystyrene-divinylbenzene (PS/DVB) [19,20]. Materials used for HPLC, like silica phases [21], are rarely adopted in IC because of their restricted pH stability.

A variety of methods exists to functionalize supporting materials either covalently or non-covalently. Non-covalent functionalization methods utilize electrostatic [[22], [23], [24], [25]] or hydrophobic [[26], [27], [28]] interactions to attach the functional groups or functional layers on the surface of the supporting material. Covalent functionalization methods either directly attach the exchanger groups onto the surface of the supporting material [29,30] or build layers containing the exchanger groups on top of the supporting material [19,20,31].

A popular way to functionalize supporting materials is the graft-polymerization that creates covalently bonded chains containing the anion exchangers on the supporting materials surface [[32], [33], [34]]. Graft-polymerization produces exchangers with pellicular structures, resulting in high separation efficiencies [5]. Schomburg et al. published the first application of graft-polymerized IC stationary phases in 1991 for cation exchangers based on polymer coated silica particles [35]. Different graft-methods like the widely-used atom transfer radical polymerization [5,[36], [37], [38]] or even free radical polymerizations [39] can be applied to create grafted ion exchangers.

### 1.1 Selectivity in anion exchange chromatography

Influencing the selectivity and maintaining high performance as well as short retention times is a demanding task. When developing ion exchangers, it is important to identify the influence of changes of the functionalization process on selectivity. To quantify differences of the selectivity, the selectivity factor *α* of two analytes is calculated from their retention factors *k.* Comparison of *α* on differently produced columns gives information about selectivity changes and how to achieve a distinct elution order of the analytes. Selectivity factor is calculated as shown in Equation (1) where k(B)>k(A).

(1) $$\alpha_{A,B}=\frac{k\left(B\right)}{k\left(A\right)}$$

The combination of this equation with the retention model for monovalent eluents [3,40,41,46] gives information on how to influence the selectivity of an exchanger. Equation (2) shows the dependency of *k* for an analyte *A* on the effective charge of the analyte *x*_*A*_ and of the eluent ion *y*, the equilibrium constant *K*_*A,E*_, the exchanger capacity *Q*, the phase-volume ratio *Φ* and lastly the eluent concentration $c\left(E^{y-}\right)$.

(2) $$k\left(A\right)={\left(K_{A,E}\right)}^{\frac{1}{y}}\cdot {\left(\frac{Q}{y}\right)}^{\frac{x_{A}}{y}}\cdot \Phi \cdot c{\left(E^{y-}\right)}^{-\frac{x_{A}}{y}}$$

Applying this to equation (1) results in the term:

(3) $$\alpha_{A,B}=\frac{{\left(K_{B,E}\right)}^{\frac{1}{y}}\cdot {\left(\frac{Q}{y}\right)}^{\frac{x_{B}}{y}}\cdot \Phi \cdot c{\left(E^{y-}\right)}^{-\frac{x_{B}}{y}}}{{\left(K_{A,E}\right)}^{\frac{1}{y}}\cdot {\left(\frac{Q}{y}\right)}^{\frac{x_{A}}{y}}\cdot \Phi \cdot c{\left(E^{y-}\right)}^{-\frac{x_{A}}{y}}}$$

This can be simplified as:

(4) $$\alpha_{A,B}={\left(\frac{K_{B,E}}{K_{A,E}}\right)}^{\frac{1}{y}}\cdot {\left(\frac{Q}{y}\right)}^{\frac{x_{B}-x_{A}}{y}}\cdot c{\left(E^{y-}\right)}^{\frac{x_{A}-x_{B}}{y}}$$

The selectivity therefore depends on *K*_*A,E*_ and *K*_*B,E*_, *Q*, $c\left(E^{y-}\right)$ as well as *x*_*A*_ and *x*_*B*_ and the charge of the eluent ions *y*. For $x_{A}=x_{B}$ the equation can be simplified further:

(5) $$\alpha_{A,B}={\left(\frac{K_{B,E}}{K_{A,E}}\right)}^{\frac{1}{y}}$$

Changes of *Q* or *c(E*^*y-*^*)* should only influence the selectivity of two analytes if their effective charges *x*_*A*_ and *x*_*B*_ differ significantly from one another. Nonetheless a possible effect of *Q* and *c(E*^*y-*^*)* on the selectivity should not be completely omitted if one wants to create exchangers with a specific selectivity.

To render the selectivity, influencing the ion exchange equilibrium constants of the analytes may be the most promising way. In reality it is the most difficult approach because one equilibrium constant has to be influenced without influencing the other in the same way, negating any effect on the selectivity. Additionally it has to be identified how exactly the equilibrium constants can be influenced. Usually the equilibrium constants are influenced by the used exchange group and by matrix effects caused by the supporting material or the eluent. For example, the choice of different ammonium-substituents results in exchangers with different selectivities [[42], [43], [44]]. In contrast changing the eluent concentration or the capacity of ion exchangers is much simpler, but not always applicable. Column capacity and *c(E*^*y-*^*)* determine the time of an analysis that should neither be too short nor too long and therefore have to be choosen accordingly. Additionally, the selectivity might not only be influenced by the ion exchange process, but also by secondary interactions of the analytes. Ion exchangers based on PS/DVB or EVB/DVB often show van-der-Waals-, π-π- or anion-π-interactions of the more polarizable anions with the polymer surface, introducing a second retention mechanism in addition to the ion exchange [5,45].

### 1.2 Radical graft-polymerization of anion exchangers

**Fig. 1:**
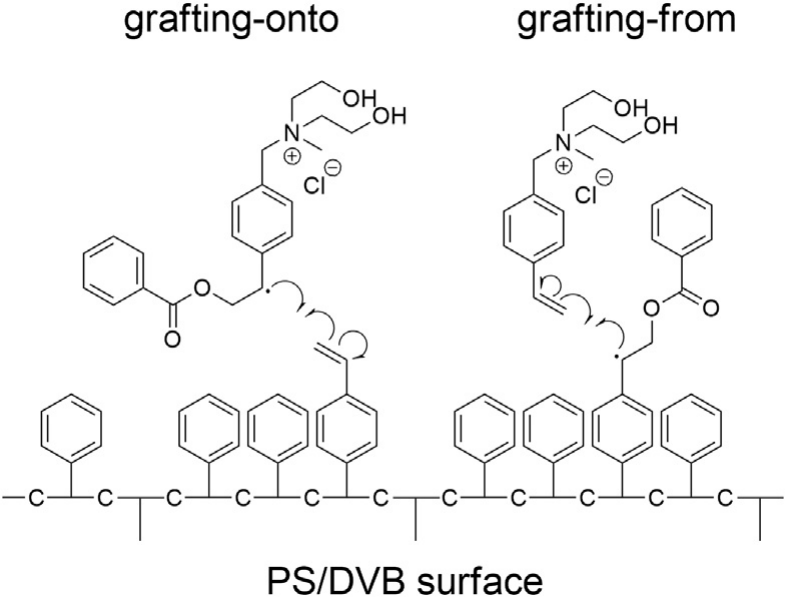
Schematic comparison of a grafting-onto and grafting-from mechanism.

In our previous work [46] we established a method to create high performance anion exchangers by functionalization of PS/DVB utilizing a graft-polymerization in which the monomer contains the exchange group. The method allows the direct functionalization of unmodified PS/DVB particles with a preformed anion exchange group attached to vinylic double bonds using remaining vinylic double bonds on the surface of highly crosslinked PS/DVB. IR spectroscopy provides a fast and simple way to differentiate between a grafting-from or grafting-onto mechanism by analyzing the percentage of converted vinylic double bonds (*DB%*) of the anion exchangers after functionalization with different amounts of monomer. The difference between grafting-from and grafting-onto mechanism is shown in Fig. 1. In case of a grafting-onto mechanism the radical initiator generates an intermediate with the monomer in free solution. This intermediate subsequently starts the grafting process on the polymer surface. In comparison the grafting-from mechanism starts with the reaction of radical initiator and polymer surface.

*DB%* can be easily calculated using the following formula using the signal at 989 cm^−1^.

(6) $$DB\%=\frac{\text{log}\left(\frac{I_{0,\;functionalized}}{I_{functionalized}}\right)}{\text{log}\left(\frac{I_{0,unfunctionalized}}{I_{unfunctionalized}}\right)}\cdot 100\%$$

A previous method [46] to create anion exchangers by graft-polymerization used potassium persulfate (KPS) as a radical initiator to graft the functional monomer N-(dihydroxyethyl)-N-methyl-N-(4-vinylbenzyl)-methyl-ammonium (VB-DEMA) onto the surface of PS/DVB particles in a mixture of water and dimethyl sulfoxide (DMSO). The reaction utilizes also the vinylic double bonds present on the monomer as well as on the PS/DVB. This combination led to a grafting-onto mechanism. The radical initiator does not interact with the vinylic double bonds on the PS/DVB surface directly. Instead a monomer-radical is generated, which is able to react with the vinylic double bonds on the PS/DVB surface to initiate the chain growth. Changes of $\alpha_{Br^{-},\;Cl^{-}}$ and $\alpha_{NO_{3}^{-},\;Cl^{-}}$, resulting in coelution with other ions like SO_4_^2−^ or PO_4_^3−^ at higher capacities, were observed when making columns with different exchange capacities leading to different retention times. Contrary to our expectations those differences in selectivity were not caused by differences in the chain length but they are depended on *Q* of the anion exchangers. Furthermore the rivaling homopolymerization process of the monomer limited the achievable capacity of the exchangers. Therefore further investigation on the understanding of selectivity changes was necessary. In this work, a different, less polar radical initiator and different organic solvents are utilized to influence the graft-mechanism and the selectivity of the prepared anion-exchangers to gain some way to control and understand the functionalization process as well as the influences on the capacity.

## 2 Experimental

### 2.1 Materials

The PS/DVB particles used as supporting material for all functionalizations were prepared according to the procedure described in Refs. [47,48] for materials with 55% nominal crosslinkage. The used polymer batches had average diameters between 4.0 and 4.5 μm and were highly monodisperse with specific surface areas *A*_*S*_ of 1000–1200 m^2^ g^−1^ and mean pore sizes *Φ*_*50*_ of 40–45 nm.

Ultrapure water was used as received from a MilliQ-system (Merck-Millipore Billerica, MA, USA). The monomer was synthesized using 4-vinylbenzyl chloride (90%, Sigma-Aldrich, Steinheim, Germany) and *N*-methyl diethanolamine (≥99% Sigma-Aldrich, Steinheim, Germany) without further treatment. The detailed procedure is given in Ref. [46]. DMSO (99.5%, Grüssing GmbH, Grüssing, Germany) and benzoyl peroxide (for synthesis, 25% water, Merck, Darmstadt, Germany) as well as the organic solvents for the synthesis were used as received.

For preparation of the eluents sodium carbonate (p.a., VWR chemicals, Leuven, Belgium) and sodium hydroxide solution (50%, p.a., Merck, Darmstadt, Germany) were used. The IC standards were prepared from the sodium salts (p.a.) of the anions.

### 2.2 Functionalization of PS/DVB particles

2.00 g of PS/DVB and 0.74 mmol of the radical initiator benzoyl peroxide were stirred at 70 °C in a mixture of one of the organic solvents in water (4.2% organic solvent, v/v) for 5 min under inert gas atmosphere. The amount of organic solvent was kept as low as possible as higher amounts result in exchangers with lower capacity. Nonetheless a certain amount of organic solvent is necessary to sufficiently wet the PS/DVB for dispersion in water. Then a monomer solution containing 0–125 mmol VB-DEMA was added to a total volume of 120 mL and the mixture stirred for 4 h at 70 °C. For stirring an overhead stirrer was used to avoid mechanical destruction of the PS/DVB particles by mechanical grinding. Finally, the polymer was filtered and washed with 100 mL acetone, water and 7.5 mmol L^−1^ Na_2_CO_3_ solution.

### 2.3 Ion chromatographic characterization

For ion chromatography the functionalized polymer was slurry-packed at 500 bar into 100 mm × 4 mm i.d. PEEK columns using a solution of 15 mmol L^−1^ Na_2_CO_3_ as the packing eluent. After 300 mL of packing eluent had passed the column the packing procedure was ended. Before measurement the column was equilibrated in 7.5 mmol L^−1^ Na_2_CO_3_ with 0.75 mmol L^−1^ NaOH for 4 h at 45 °C.

The IC measurements were executed using a Metrohm 850 Professional IC with 766 IC Sample Processor. For separation an eluent of 7.5 mmol L^−1^ Na_2_CO_3_ with addition of 0.75 mmol L^−1^ NaOH was used at 0.8 mL/min. The column temperature was set to 45 °C. A mixture of 2 mg L^−1^ F^−^ and Cl^−^, 5 mg L^−1^ NO_2_^−^, 10 mg L^−1^ Br^−^, NO_3_^−^ and SO_4_^2−^ and 20 mg L^−1^ PO_4_^3−^ as well as 10 mg L^−1^ single-standards were used for characterization of the column, using sample volumes of 20 μL.

All selectivity factors were calculated using Cl^−^ as the reference ion. Shown data of asymmetry factors and theoretical plate numbers were calculated by MagIC Net 3.0 using the USP formulas. The retention factors were calculated taking the system volume into account by subtracting the system time without a column from both hold-up time and retention time.

### 2.4 Polymer characterization

Polymer samples were washed with water to remove remaining Na_2_CO_3_ and then dried for two days at 60 °C. The dry polymers were then analyzed directly using the UATR two unit of a Perkin Elmer Spectrum two IR spectrometer. For elemental analysis of the polymers, the sample was washed repeatedly with water and then dried for 2 days at 60 °C. The samples were then analyzed by elemental analysis using a vario MICRO cube by Elementar for carbon, hydrogen, nitrogen and sulfur and a rapid OXY cube by Elementar for oxygen.

The exchanger capacity *Q* can be directly calculated from the nitrogen amount of the polymer, assuming all exchanger groups contribute to the ion exchange process.

### 2.5 Approximation of benzoyl peroxide (BPO) solubility

For approximation of the solubility of BPO in the different organic solvents during the functionalization process an excess of BPO was added to the particular organic solvent. The mixture was treated with ultrasound at room temperature for 4 h. To quantify the amount of solved BPO a HPLC method based on Ref. [49] was used. The sample was diluted in acetonitrile and measured with an eluent of 55% acetonitrile and 45% water (v/v) at a flow of 1 mL/min. The thermostat was set to 40 °C and a detection wavelength of 195 nm was used. The measurements were conducted three times for each organic solvent and the average calculated.

HPLC measurements were performed using a Hewlett Packard 1100 Binary Pump, 1100 Autosampler and 1100 Column Thermostat, an Agilent 1100 DAD, an ERC-3315 degasser by ERC and a Discovery C18, 4 mm i.d. column by Supelco.

### 2.6 Testing the polymer stability

To verify a covalent functionalization on the exchangers the created columns were washed with 10% acetone solution in water at 0.3 mL/min and 45 °C for at least 16 h. Decreased retention times after the washing step indicates the removal of homopolymer.

## 3 Results and discussion

### 3.1 Radical initiator benzoyl peroxide

Anion exchangers of varying capacity were prepared by graft-polymerization of VB-DEMA onto the surface of PS/DVB using benzoyl peroxide as the radical initiator. The created columns are directly compared to those of the exchangers functionalized with KPS from Ref. [46].

**Fig. 2:**
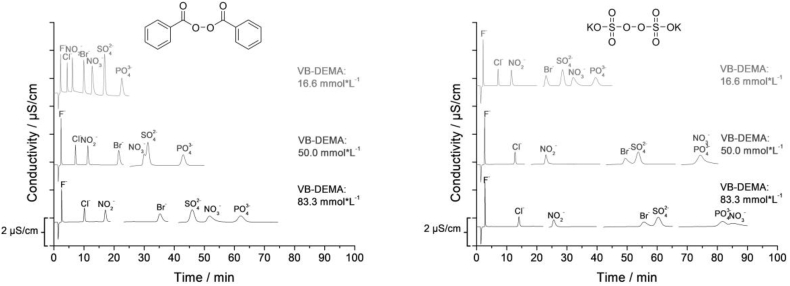
Comparison of the chromatograms of anion exchangers functionalized with benzoyl peroxide (left) and potassium persulfate (right) using equal concentrations of monomer.

As can be seen in Fig. 2 the functionalization with BPO was successful, but the anion-exchangers show lower capacities than those made with KPS when using same concentration of VB-DEMA. In both cases, the exchangers show changes of the elution order with increasing capacity. When using 16.7 mmol L^−1^ VB-DEMA with BPO, NO_3_^−^ still elutes in front of SO_4_^2−^. Using 50.0 mmol L^−1^ VB-DEMA with BPO results in a coelution of those anions and in case of 83.3 mmol L^−1^ NO_3_^−^ shows higher retention times than SO_4_^2−^. The columns functionalized with BPO possess the same high chromatographic performance as those functionalized with KPS.

According to the retention mechanism one would expect the opposite observation: Increasing the capacity should result in a stronger retention of multiply charged ions, like SO_4_^2−^, in comparison to single charge ions like Br^−^ or NO_3_^−^. The gap between those signals should widen and no change of the elution order should be observed. It is therefore unlikely that the observed change in selectivity is caused by the capacity. Instead either the exchanger affinity is influenced with increasing capacity or another retention mechanism gains importance with increasing capacity of the exchanger.

The capacity of the column Q increases linearly with increasing amount of VB-DEMA when utilizing BPO, whereas for KPS a maximum of the capacity is achieved at 6 mmol VB-DEMA. As discussed in Refs. [46,50] using KPS as a radical initiator, the homopolymerization of VB-DEMA rivals the graft-process strongly. At higher concentrations of VB-DEMA the homopolymerization is preferred over the functionalization. This effect cannot be observed for BPO and indicates a grafting-from instead of a grafting-onto mechanism.

As shown in Ref. [46] distinguishing a grafting-onto mechanism from a grafting-from mechanism is possible by utilizing IR spectroscopy and analyzing *DB%* at different amounts of VB-DEMA. If the amount of VB-DEMA does not influence the amount of double bonds converted in the graft-process, a grafting-from mechanism takes place, whereas an influence indicates a grafting-onto mechanism. A grafting-from mechanism should also suppress the homopolymerization, as the radicals are generated on top of the PS/DVB surface and no monomer-radicals are generated by the radical initiator.

**Fig. 3:**
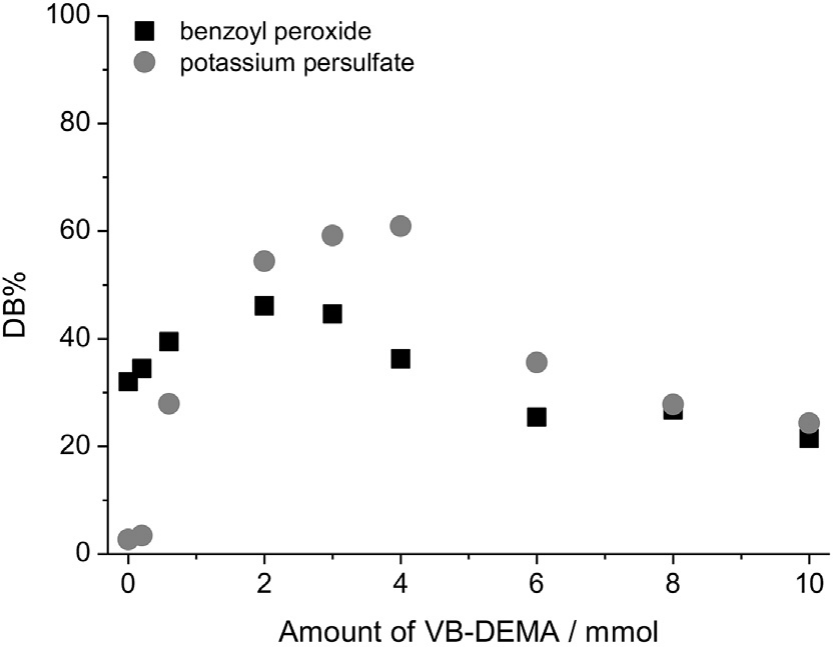
Percentages of converted double bonds after functionalization with different amounts of monomer VB-DEMA for the radical initiators benzoyl peroxide and potassium persulfate.

Fig. 3 shows DB% for the anion exchangers functionalized with different amounts of VB-DEMA when utilizing BPO and KPS. Even if there is no VB-DEMA used, 32% of the vinylic double bonds are converted when using BPO, whereas KPS shows no conversion without VB-DEMA. This indicates the presence of a grafting-from mechanism when using BPO. Instead of a constant DB% when increasing the amount of VB-DEMA for BPO a slight increase of DB% up to 46% at 2 mmol VB-DEMA can be observed. Increasing the VB-DEMA amount further results in a decreasing DB% to 21% at 10 mmol VB-DEMA. Therefore a mixed reaction mechanism is present where both grafting-onto and grafting-from take place when utilizing BPO as the radical initiator. The decrease of DB% at amounts of VB-DEMA higher than 2 mmol can therefore also be explained by an ongoing homopolymerization as a byproduct of the grafting-onto mechanism.

**Fig. 4:**
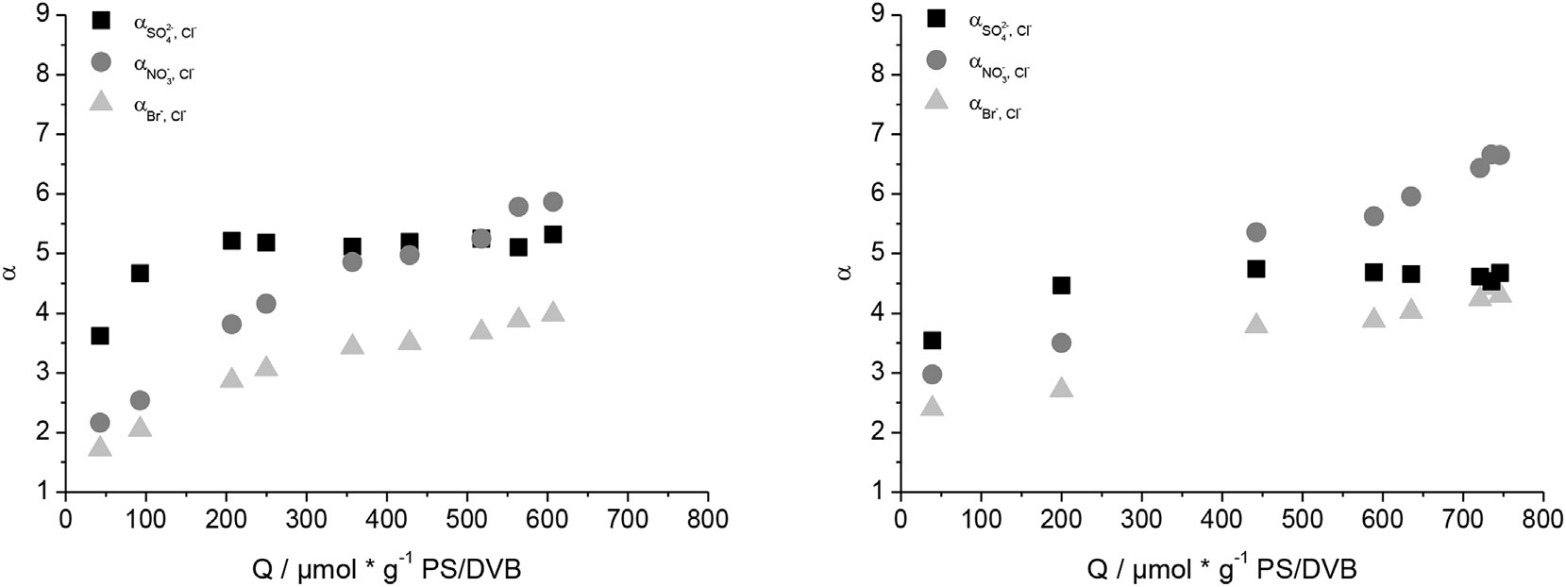
Comparison of the selectivity factors of Br^−^, NO_3_^−^ and SO_4_^2−^ in reference to Cl^−^ of columns made with benzoyl peroxide (left) and potassium persulfate (right) as radical initiator.

Even though the change of the radical initiator to BPO influences the graft-mechanism and results in a linear correlation of the capacity and the concentration of monomer used, the columns functionalized with BPO show a similar behavior of the selectivity factor for Br^−^, NO_3_^−^ and SO_4_^2−^ with increasing capacity when compared to columns functionalized with KPS. Fig. 4 shows an increase of Br^−^ and NO_3_^−^ selectivity factors at high capacities, whereas SO_4_^2−^ remains unaffected. The increase of Br^−^ and NO_3_^−^ selectivity factors is slightly stronger when using KPS as the radical initiator, indicating a small effect of the BPO on the selectivity. Changing the radical initiator alone is neither sufficient to prevent the grafting-onto mechanism, nor to influence the selectivity in the desired way.

### 3.2 Solubility of benzoyl peroxide in organic solvents

Another way to influence the mechanism of the functionalization and the resulting selectivity of the prepared columns is a change of the organic solvent. The PS/DVB particles cannot be dispersed in pure water due to the difference in polarity. Therefore an organic solvent is used to wet the polymer and make it dispersible in water. This means the radical initiator does not only need to have a high affinity towards the PS/DVB particle but also towards the organic solvent. The higher the solubility of the radical initiator in the organic solvent, the better the PS/DVB surface should be available to it. Furthermore the concentration of organic solvent should be higher in close proximity to the PS/DVB surface than in the free solution consisting mainly of water. Under those circumstances, a hydrophobic radical initiator should reside primarily close to the PS/DVB surface. The radicals are therefore generated in close proximity to the PS/DVB surface to initiate a grafting-from- instead of a grafting-onto mechanism or the homopolymerization.

To be applicable in the graft-functionalization the organic solvent needs to fulfill certain conditions. It must be sufficiently water soluble to allow a 4.2% organic solvent fraction in water (v/v). The required amount of radical initiator has to be soluble in the solvent. And last but not least the solvents needs to have a sufficiently high boiling point to avoid solvent loss during the functionalization.

Including DMSO, thirteen suitable organic solvents were investigated in the graft-polymerization step and their effect on the resulting anion-exchangers were examined. The solubility of BPO in those solvents was approximated by HPLC measurements of BPO solutions in the particular solvent after 4 h treatment with ultrasound at room temperature. Table 1 shows the calculated approximated solubility of BPO *L(BPO)* in the particular solvent. *L(BPO)* ranges from 138 g L^−1^ for 3-Pentanone to 32.6 g L^−1^ for 1-Methoxy-2-propanol. BPO has a low solubility in DMSO of approximately 40.0 g L^−1^. No solvents were found with *L(BPO)* between 50.0 g L^−1^ and 90.0 g L^−1^.

**Table 1:** Approximated solubility of benzoyl peroxide in different organic solvents.

Solvent	L(BPO) [g·L^−1^]	Solvent	L(BPO) [g·L^−1^]
Acetyl acetone	107	1-Methoxy-2-propanol	32.6
Benzyl alcohol	48.7	1-Methoxy-2-propylacetate	111
Cyclohexanone	128	2-Methoxyethyl acetate	92.6
Diacetone alcohol	38.6	Methylisobutylketone	135
Dimethyl sulfoxide	40.0	3-Pentanone	138
2-Ethoxyethyl acetate	108	Propylene carbonate	46.9
Ethylene carbonate	43.0		

### 3.3 Influence of initiator solubility

**Fig. 5:**
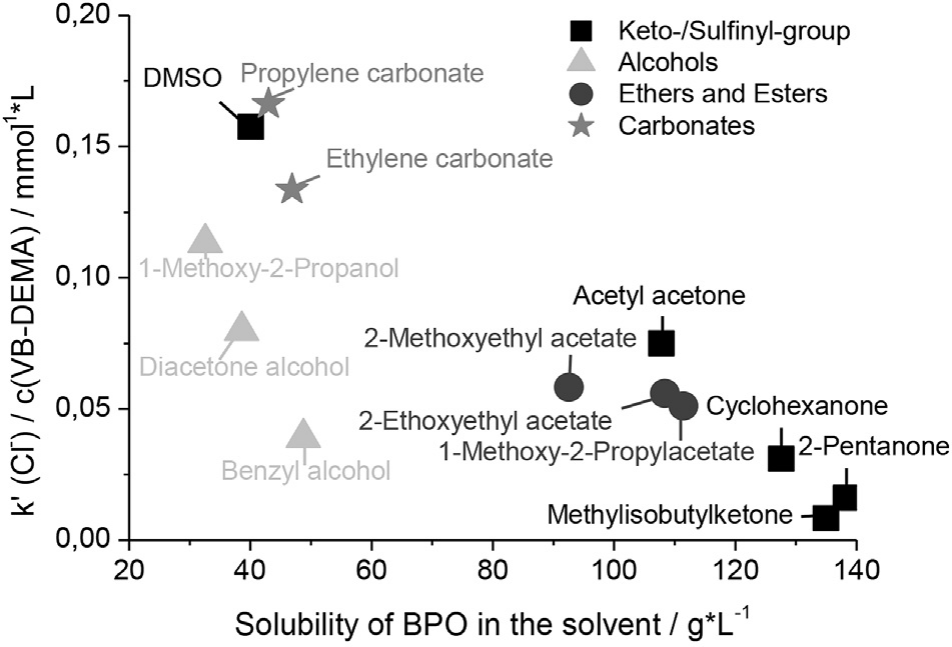
Influence of the solubility of BPO on the generated capacity.

When using the different solvents with different solubilities for the radical initiator *L(BPO)* in the graft-polymerization with a monomer concentration of 16.7 mmol L^−1^ VB-DEMA columns with varying capacity are generated. Organic solvents with high *L(BPO)* produced columns with very low capacities, requiring higher amounts of VB-DEMA to allow a separation of the seven anions. Fig. 5 shows the influence of *L(BPO)* on the capacity generated, the capacity is represented by *k(Cl*^*−*^*)* divided by the concentration of VB-DEMA used for functionalization. At *L(BPO)* < 60 g L^−1^ two groups of solvent can be observed. Solvents with alcohol groups produce columns with significantly lower capacity than solvents with comparable *L(BPO)* but without an alcohol group. At *L(BPO)* > 90 g L^−1^ a linear decrease of the achieved capacity is observed with increasing *L(BPO)*. Excluding the organic solvents with alcohol groups shows a linear correlation between *L(BPO)* and the generated capacity.

Comparing the Br^−^, NO_3_^−^ and SO_4_^2−^ selectivity factors of columns with similar *k(Cl*^*−*^*)* from 1.0 to 2.0 shows no influence of *L(BPO)*. This can easily be explained by our previous results. At low column capacities we obtained similar results for columns functionalized with BPO and KPS. The differences appear at higher column capacities.

Therefore to investigate the effect of the solvent more in depth, it is necessary to create columns of varying capacity, to identify the ongoing graft-mechanism and to investigate the influence of the capacity on the selectivity. For this columns of varying capacity were functionalized with cyclohexanone as the organic solvent. Cyclohexanone features a high *L(BPO)* in contrast to DMSO but allows to create columns with higher capacity at lower amounts of VB-DEMA than 2-pentanone or methyl isobutyl ketone. For functionalization varying amounts of VB-DEMA ranging from 0 to 30 mmol were used with cyclohexanone as the organic solvent and BPO as the radical initiator. Comparing the results of DMSO with those of cyclohexanone should allow a better understanding on the influence of the organic solvent.

**Fig. 6:**
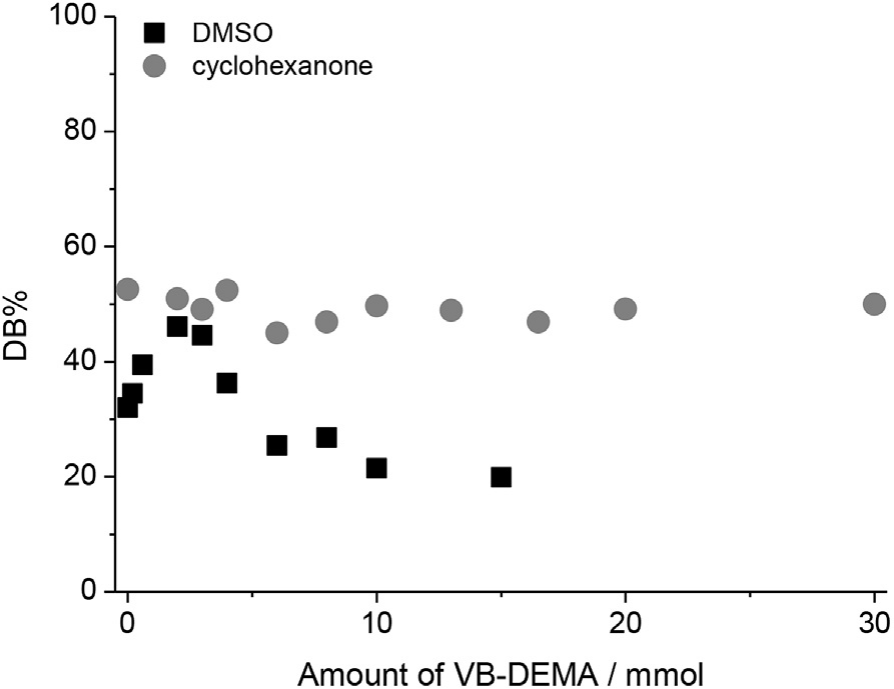
Influence of the used amount of VB-DEMA on the amount of converted double bonds.

First of all we investigated the reaction mechanism. Using DMSO and BPO shows a mixed mechanism of both grafting-onto and grafting-from. As *L(BPO)* is much higher in cyclohexanone, a stronger influence of the grafting-from mechanism is to be expected. Fig. 6 shows DB% for columns functionalized with DMSO and cyclohexanone where different amounts of VB-DEMA were used. The results conform to the expectation of higher *L(BPO)* favoring a grafting-from mechanism. Using cyclohexanone DB% stays at about 50% regardless of the amount of VB-DEMA used.

**Fig. 7:**
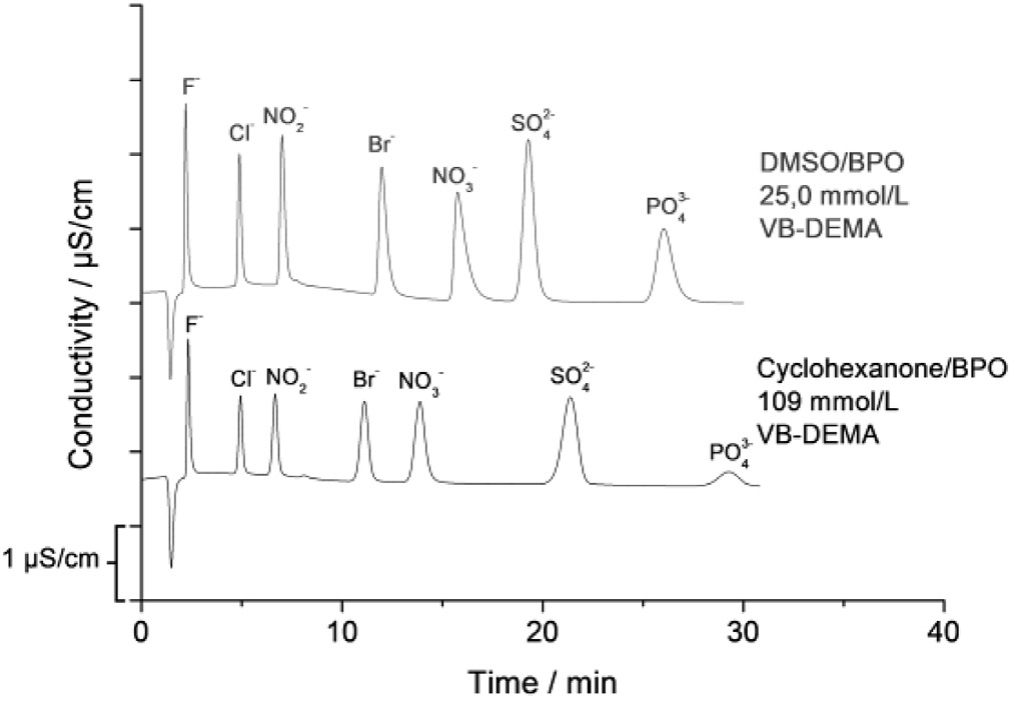
Chromatograms of anion-exchangers with similar capacities functionalized with DMSO and cyclohexanone.

Additionally the exchangers functionalized with cyclohexanone show different selectivity and performance in comparison to those functionalized with BPO. Fig. 7 compares the chromatograms of two columns functionalized with DMSO and with cyclohexanone. Both exchangers show similar *Q* but different selectivities for Br^−^ and NO_3_^−^ as well as different performance. The column functionalized with DMSO has a high performance with theoretical plate number of 60000 m^−1^ for SO_4_^2−^, whereas the column functionalized with cyclohexanone shows broader peaks and lower theoretical plate number of 37000 m^−1^ for SO_4_^2−^. The same behavior can be observed for columns with higher and lower capacity.

**Fig. 8:**
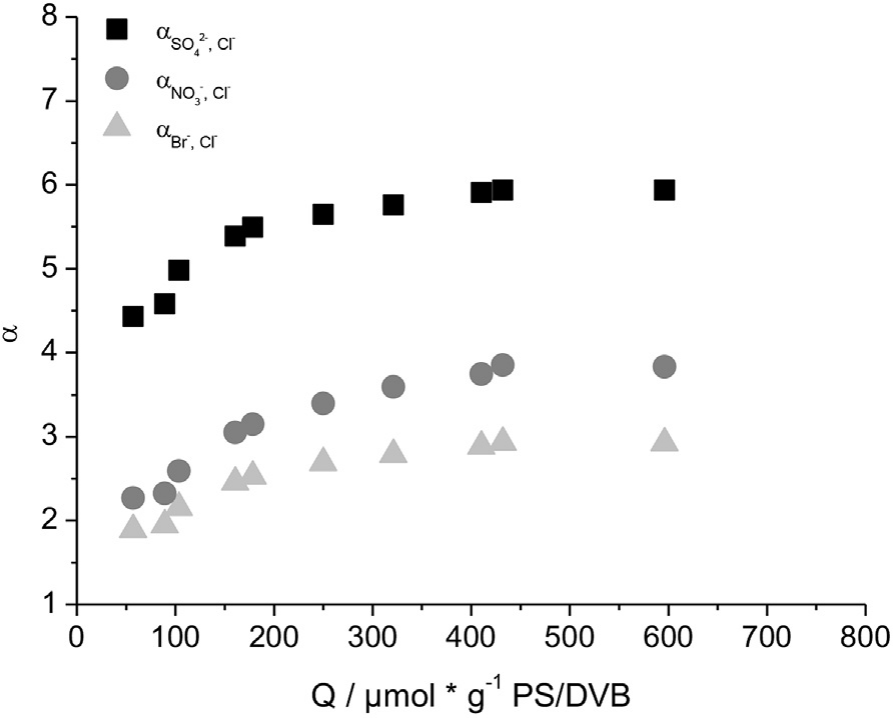
Selectivity factors for Br^−^, NO_3_^−^ and SO_4_^2−^ in reference to Cl^−^ of columns created with cyclohexanone at different column capacities.

The selectivity factors ${\text{α}}_{Br^{-},\;Cl^{-}}$ and ${\text{α}}_{NO_{3}^{-},\;Cl^{-}}$ are lower when using cyclohexanone, successfully influencing the selectivity in the desired way. Plotting ${\text{α}}_{Br^{-},\;Cl^{-}}$, ${\text{α}}_{NO_{3}^{-},\;Cl^{-}}$ and ${\text{α}}_{SO_{4}^{2-},\;Cl^{-}}$ against *Q* in Fig. 8, shows an increase of all three selectivity factors up to *Q* of 400 μmol g^−1^. Increasing the capacity further does not influence the selectivity factors any more. In comparison to DMSO no coelution of NO_3_^−^ and SO_4_^2−^ can be achieved by simply increasing the capacity of the column. BrO_3_^−^, on the other hand, is not influenced by the capacity when using cyclohexanone.

**Fig. 9:**
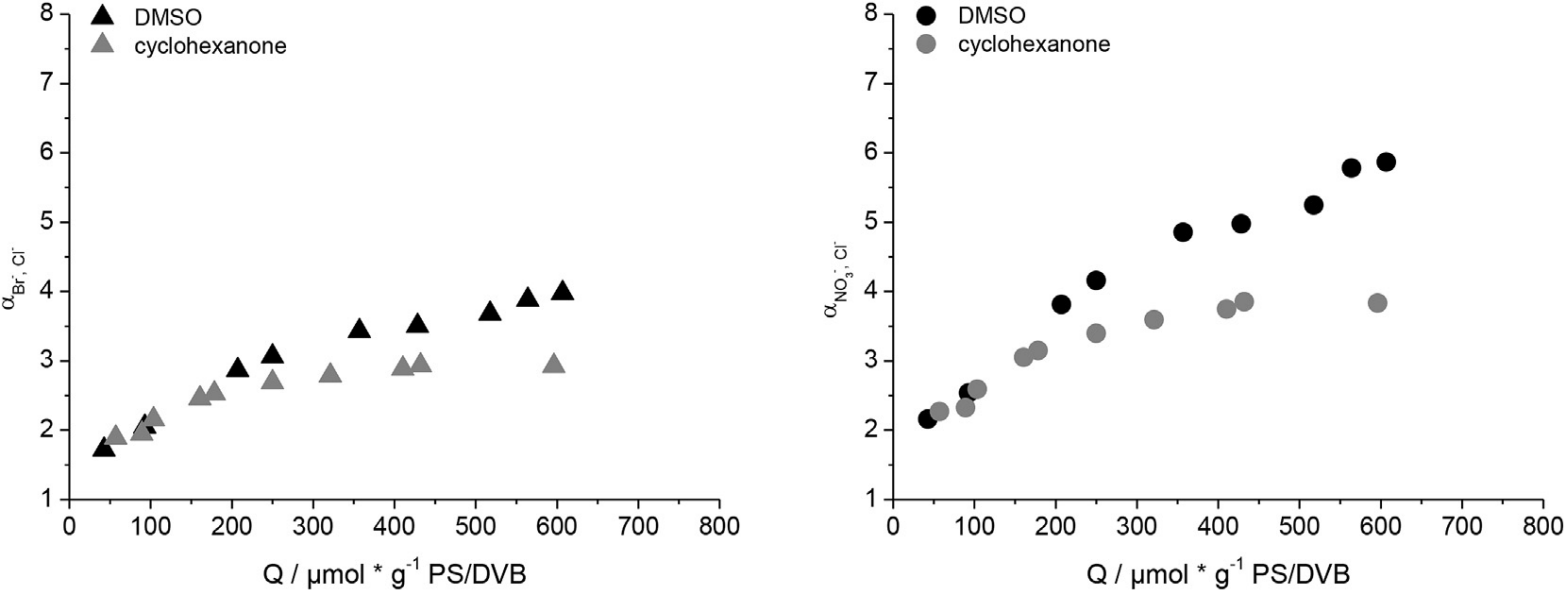
Plots of the selectivity factor of Br^−^ (left) and NO_3_^−^ (right) against the retention factor of Cl^−^ for columns functionalized with DMSO and cyclohexanone as the organic solvent.

Those results seem to contradict previous results where no difference in the Br^−^ and NO_3_^−^ selectivity factors of columns created with different organic solvents was observed. A direct comparison of the selectivity factors of Br^−^ and NO_3_^−^ for DMSO and cyclohexanone columns at different capacities, as shown in Fig. 9, explains those results. Columns with *Q* < 200 μmol g^−1^ show identical α for both Br^−^ and NO_3_^−^. Only if the capacities are increased further, differences in the selectivity become visible. The previously compared anion-exchangers with *k(Cl*^*−*^*)* ranging from 1.0 to 2.0 therefore showed comparable selectivity factors for Br^−^ and NO_3_^−^, even though different organic solvents were used.

Even though these results clearly show the selectivity of the graft-functionalized anion exchangers can be influenced by means of the used organic solvent, it is unclear what causes those differences. The graft-mechanism can most likely be excluded as a possible cause for the varying selectivities. Firstly the chemical structure of the anion exchangers should not differ greatly for the different mechanisms. Secondly the exchangers functionalized with KPS and BPO in DMSO show different reaction mechanisms but very similar selectivities. Therefore the selectivity must be influenced by some other factor. The most probable reason is the influence of side reactions. It might be possible for the organic solvent to interact with the radicals and alter the functionalization of the polymer. Furthermore the radical initiator BPO may oxidize the PS/DVB surface in addition to initiating the graft process, altering the polymer surface. Increasing solubility of BPO in the organic solvent might cause more BPO in close proximity to the PS/DVB surface and therefore an increase of surface oxidation, possibly explaining the change of selectivity.

## 4 Conclusions

Alteration of the graft-mechanism and the selectivity of graft-functionalized anion-exchangers were possible simply by a change of the radical initiator and the organic solvent. Using BPO instead of KPS resulted in anion-exchangers with similar high performance and selectivity, but influenced the graft-mechanism strongly. The higher affinity of BPO towards the hydrophobic PS/DVB surface allowed a grafting-from mechanism to take place and resulted in a linear increase of the capacity with *c(VB-DEMA)*, whereas functionalization with KPS showed a saturation-like behavior. However when using DMSO as the organic solvent the grafting-onto mechanism and the homopolymerization were still taking place next to the grafting-from mechanism. Using organic solvents with a higher solubility of the radical initiator allowed elimination of the grafting-onto mechanism and the homopolymerization. At the same time organic solvents, like cyclohexanone, with a higher solubility of BPO produced anion-exchangers with differing selectivity for Br^−^, NO_3_^−^ and BrO_3_^−^, lower performance and capacity if the same amount of monomer is used. The selectivity factor of BrO_3_^−^ versus Cl^−^ decreases linearly with increasing solubility of BPO and becomes independent from the exchanger capacity. In contrast differences of the Br^−^ and NO_3_^−^ selectivity factors are only visible for exchangers with higher capacity and *k(Cl*^*−*^*)* > 3. The cause for those differences in selectivity is unclear as a change of the graft-mechanism should not change the chemical structure of the exchanger much.

Further studies should investigate the source of the selectivity changes of the grafted anion-exchangers as well as a way to optimize the graft-functionalization when utilizing organic solvents with high solubility of BPO.
