# Supplementary material for: Influencing the selectivity of grafted anion exchangers utilizing the solubility of the radical initiator during the graft process
Source: Anal Chim Acta X. 2019 May 23;2:100019. doi: 10.1016/j.acax.2019.100019 (PMC7587026; doi:10.1016/j.acax.2019.100019)
Supplement: Multimedia component 1 [file mmc1.docx]

1. **Supplementary Information ACA-19-563R1**

**A.1. Relation between the amount of monomer and column capacity *Q* for two different radical initiators**

The influence of the amount of monomer on the column capacity *Q* when using the different radical initiators potassium peroxide and benzoyl peroxide is shown in Figure S1. The capacity of the column *Q* increases linearly with increasing amount of VB‑DEMA when utilizing BPO, whereas for KPS a maximum of the capacity is achieved at 6 mmol VB-DEMA. As discussed in Refs. [46] and [50] using KPS as a radical initiator, the homopolymerization of VB‑DEMA rivals the graft-process strongly. At higher concentrations of VB‑DEMA the homopolymerization is preferred over the functionalization. This effect cannot be observed for BPO and indicates a grafting-from instead of a grafting-onto mechanism.

Figure S1: Comparison of the influence of the VB-DEMA concentration on the retention times of chloride when using benzoyl peroxide or potassium persulfate as radical initiator.

**A.2. Relation between the amount of monomer and column capacity *Q* for two different solvents**

Figure S2 compares the resulting *Q* of columns functionalized with different amounts of VB‑DEMA when using DMSO or cyclohexanone as the organic solvent. At the same VB‑DEMA amounts DMSO generates columns with higher capacity than cyclohexanone. As higher amounts of VB‑DEMA were with cyclohexanone we also increased the amount of VB‑DEMA using DMSO up to 15 mmol. Using cyclohexanone shows a linear correlation up to 30 mmol VB‑DEMA used, whereas further increase of the VB‑DEMA amount to 15 mmol with DMSO shows that the linear correlation is limited to lower amounts of VB‑DEMA. This is in line with our previous observation of both grafting-onto and grafting-from mechanism being present when using DMSO.

**Figure S2****: Influence of the used concentration of VB‑DEMA on the retention factor of Cl^-^ of the resulting anion exchanger.**

**A.3. Selectivity changes for selected ions depending on BPO solubility in the solvent**

Figure S3 shows the minor $\alpha_{{Br}^{-}, {Cl}^{-}}$ and $\alpha_{{NO}_{3}^{-}, {Cl}^{-}}$ decrease with increasing *L(BPO)*. In comparison a small increase in $\alpha_{{SO}_{4}^{2-}, {Cl}^{-}}$ is visible for solvents with high *L(BPO)*. Whereas those selectivity factors are influenced only slightly, the selectivity factor of bromate, another polarizable anion, changes more clearly. Additionally with increasing *L(BPO)* a linear decrease of $\alpha_{{BrO}_{3}^{-}, {Cl}^{-}}$ is observed.

**Figure S3: Influence of the solubility of BPO on the selectivity factors of Br^-^, NO_3_^-^ and SO_4_^2-^.**

The use of organic solvents with different solubility of the radical initator BPO results in different selectivities for BrO_3_^-^. A linear decrease of $\alpha_{{BrO}_{3}^{-}, {Cl}^{-}}$ is observed in Figure S4 for columns functionalized with organic solvents with increasing solubility of BPO.

Figure S4: Influence of the solubility of BPO on the selectivity factors BrO_3_^-^.

**A.4. Selectivity changes for BrO_3_^-^/Cl^-^ with capacity *Q* for two different solvents**

Comparing the BrO_3_^-^ selectivity (expressed as selectivity factor with Cl^-^) in Figure S5, the use of cyclohexanone as the organic solvent results in selectivity factors unaffected by the column capacity, whereas the selectivity factors do increase for columns functionalized with DMSO as the organic solvent. This change of selectivity is sufficient to be visible in the chromatograms. Figure S6 compares the chromatograms of a DMSO and a cyclohexanone column with similar capacity for the seven standard anions and BrO_3_^-^. In case of the DMSO column BrO_3_^-^ coelutes with NO_2_^-^_,_ whereas it elutes directly in front of NO_2_^-^ for the cyclohexanone column.

Figure S5: Comparison of the selectivity factors for BrO_3_^-^ in reference to Cl^-^ of columns created with DMSO and cyclohexanone at different column capacities.


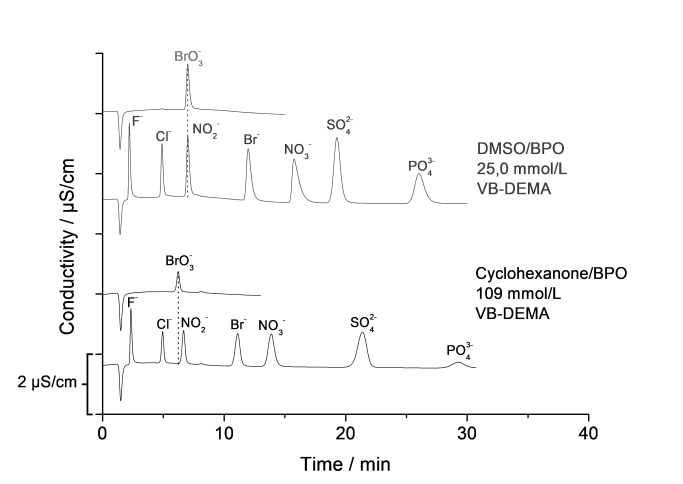


Figure S6: Chromatograms of the seven anorganic anions and BrO_3_^-^ of anion-exchangers with similar capacities functionalized with DMSO and cyclohexanone.
